# Cell death-based treatment of childhood cancer

**DOI:** 10.1038/s41419-017-0062-z

**Published:** 2018-01-25

**Authors:** Mike-Andrew Westhoff, Nicolas Marschall, Michael Grunert, Georg Karpel-Massler, Stefan Burdach, Klaus-Michael Debatin

**Affiliations:** 1 Department of Pediatrics and Adolescent Medicine, Ulm University Medical Center, Ulm, Germany; 2 Department of Radiology, German Armed Forces Hospital of Ulm, Ulm, Germany; 3 Department of Neurosurgery, Ulm University Medical Center, Ulm, Germany; 4 Department of Pediatrics and Children’s Cancer Research Center (CCRC), Technische Universität München (TUM), Munich, Germany

## Abstract

Any therapy that aims at eradicating a cancerous growth will have at its core a cell death-inducing component. Here we argue that paediatric oncology presents with its unique set of considerations and problems, which—while taking the lead from oncological research experiences obtained from the adult population—need to be clinically evaluated independently. This is particularly true when considering long-term side effects. Precision medicine offers a promising new approach in therapy, but given as a monotherapy and in a limited combination, as found in an apoptosis inducer/sensitiser combination, it will most likely lead to mutation escape of the target cell population and the emergence of resistance. However, using the increasing amount of the molecular data as the basis for a complex combination therapy combining several key components such as cell death-inducing agents, kinase inhibitors and BH3 mimetics, holds great promise.

## Facts

- Paediatric, adolescent and adult cancers are different groups of diseases occurring in distinct environments.
- Survival rates of patients afflicted with different paediatric malignancies have increased significantly over the last decades.
- Long-term complications in childhood cancers are complex and need to be carefully evaluated.
- More clinical trials are needed that focus exclusively on paediatric tumours.
- Precision medicine has so far not been shown to be more effective than “doctor’s choice”.

## Open questions

- How best to collect, identify and analyse the relevant data on patients and disease?
- How best to minimise and react to emerging long-term complications of novel therapeutic approaches?
- How best to design a precision + approach that further increase treatment efficacy while reducing negative side effects?

## Introduction

Modern cancer therapy is built on three pillars, surgery, radio- and chemotherapy, with a fourth—which actually precedes the latter two—currently gathering momentum: immunotherapy^1–3^. Surgery aside, all of these medical approaches are based on the induction of cell death, and they work. For example, the 10 years or more survival rate in the UK has more than doubled in the last 40 years and is now ~50%, although with large tumour-specific variations, ranging from 98% survival for prostate to 1% for pancreatic cancer^4^.

There are currently more than a dozen forms of cell death recognised. However, the preferred mode by which cell death is therapeutically induced remains a mechanism termed ‘apoptosis’, which also plays a crucial role in normal physiology and was first described more than 40 years ago^1,5^. Other forms of cell death that are of therapeutic interest are 'mitotic catastrophe', which is induced by some subgroups of chemotherapeutic reagents, 'autophagic cell death', which is closely related to 'autophagy', a cellular survival mechanism, and the unregulated cell death 'necrosis', which is immunogenic, i.e., causes inflammation and can elicit an immune response of potentially therapeutic value^1,6,7^.

The primary aim of cancer therapy has hardly changed since the first recorded cases of treating tumours of the breast 3500 years ago^1^, c*aedite eos* - eradicating all tumour cells and thereby curing the patient. Only if the primary aim is unachievable, would we consider minimising tumour spread and rate of growth as to achieving maximal quality and quantity of life for the patients. However, both the means of reaching that goal and our understanding of what we are fighting have changed drastically in the last two and a half decades. From a dose-escalating arms race until the maximal tolerated doses (MTDs) are reached, and, thus, accepting severe and, in extremis, lethal side effects, the focus has shifted to a metronomic chemotherapy protocol, i.e., the continuous or more frequent administration of lower therapeutic doses^8^. In addition, the increasing acceptance of the cancer stem cell hypothesis, although at best still a large simplification of biological reality^9^, has led to a shift in our understanding of tumour organisation. We no longer view cancer as an egalitarian collective of malignant cells, but understand that a hierarchical order exists, leading to a complex ecosystem of supporting and competing populations, not all of which are necessarily comprised of malignant cells^10^.

**Fig. 1:**
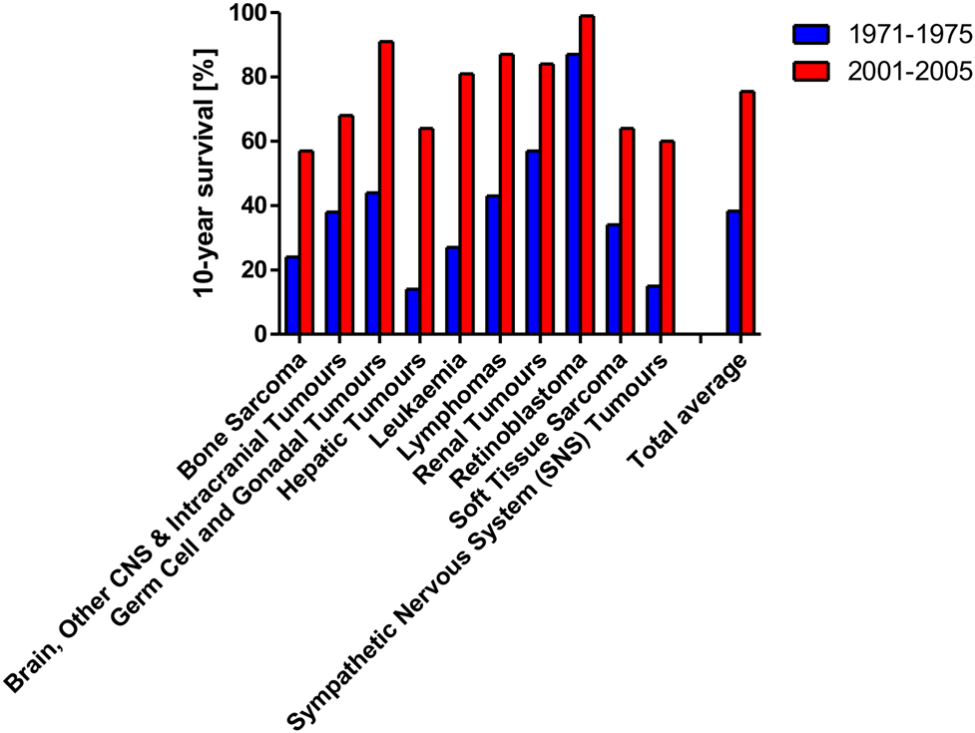
Comparison 10-year survival rates 1971–1975 vs. 2001–2005, grouped according to various childhood cancers Shown is the percentage of patients suffering from ten common childhood cancers either in 1971–1975 or 2001–2005 who survived for 10 years or more, i.e., are considered cured. The data was obtained from Cancer Research UK (www.cancerresearchuk.org/health-professional/cancer-statistics) and based on National Cancer Intelligence Network. National Registry of Childhood Tumours Progress Report, 2012. Oxford: NRCT; 2013 and 10-year actuarial survival, children aged 0–14 years, Great Britain, the 1971–2005 data were provided by Charles Stiller at the National Registry of Childhood Tumours on request in 2013

The treatment of paediatric malignancies has been one of the few great success stories in oncology. The survival rates for a wide range of childhood cancers has drastically improved over the last 40 years (Fig. 1). Childhood leukaemia 10-year survival went from 27 to 81% in a period of 30 years, while during the same time span sympathetic nervous system tumour survival went from 15 to 60%. Even already good survival rates could be further improved, for example, going from 87 to 99% for retinoblastoma. Looking at 10 common childhood malignancies the average survival rate almost doubled (a 1.9-fold increase) from the early 1970s to the early 2000s (Fig. 1). There are, nevertheless, subgroups of paediatric malignancies that we cannot control as well, for example, 2–3% of childhood acute lymphoblastic leukaemia (ALL) are refractory at presentation, while 15% relapse reducing long-term survival to 40–50%, thus, making recurrent ALL one of the leading causes of cancer-related death in children and highlighting the need for novel therapeutic approaches^11^. Similarly, Ewing’s Sarcoma has a long-term survival rate of ~75%, which is reduced to <30% when the disease has metastasised, which is the case in 20–30% of all children at clinical presentation^12^, while other paediatric cancers generally present with a dismal diagnosis such as diffuse intrinsic pontine glioma, which is associated with an overall survival of just 8–10 months^13^. While a better fundamental understanding of these treatment-resistant malignancies is needed to improve treatment options, it is also the increasing awareness of the side effects of existing (and successful) therapeutic interventions that drives current research.

## A different group of diseases?

**Fig. 2:**
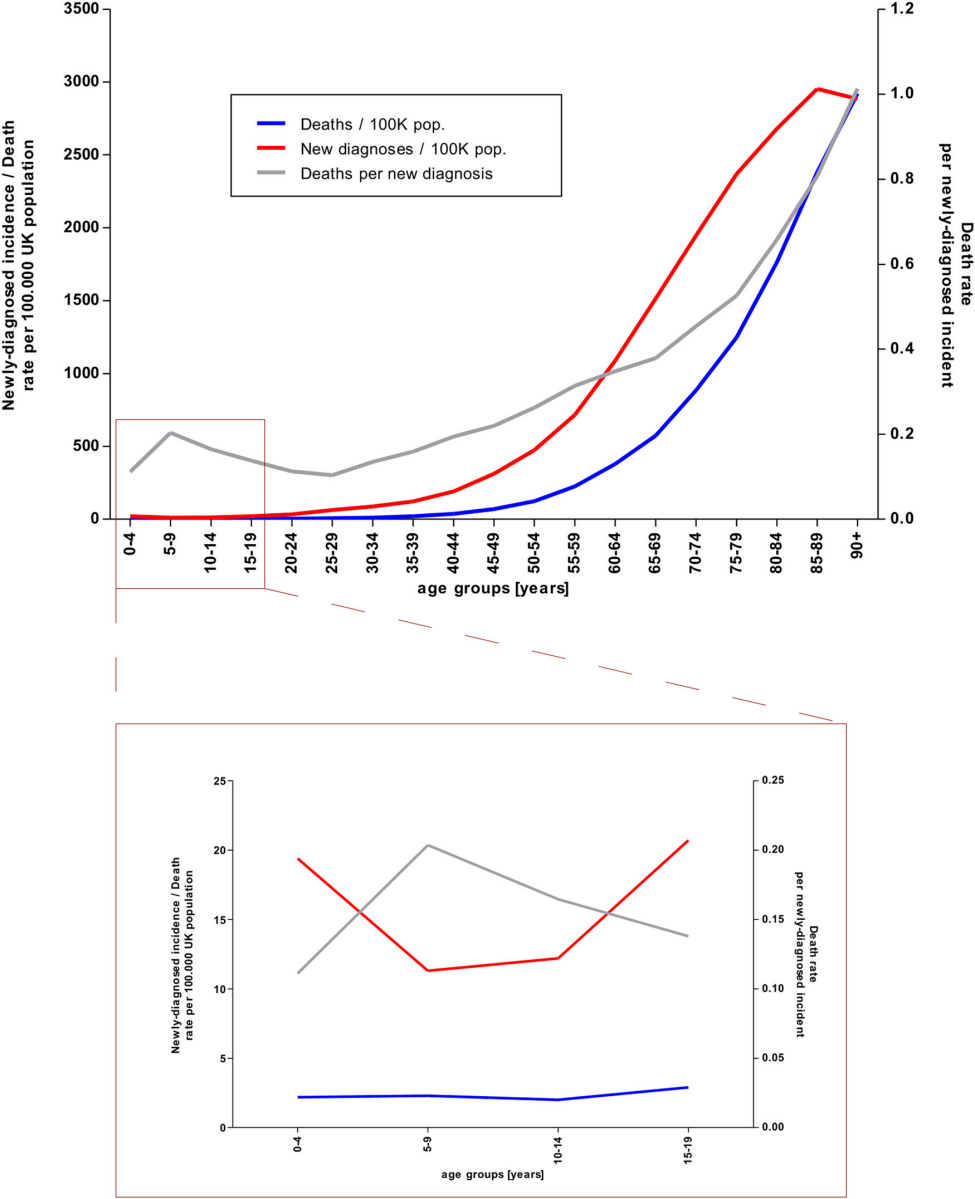
Comparison of the cancer incidence and cancer death rate between age groups Shown are the cancer-related deaths (blue line), new diagnoses of cancer (red line) and the deaths per newly diagnosed cancer (grey line) for various age groups within the UK population.The scale to the left refers to the red and blue line, the scale on the right corresponds to the grey line. The area boxed red in the upper panel is blown up in the lower panel.Of note, new diagnoses exclude non-melanoma skin cancers as there are no sufficient records.Data were obtained from Cancer Research UK (www.cancerresearchuk.org/health-professional/cancer-statistics)

Ralph Waldo Emerson once put forward that “[a]ll diseases run into one, old age”. This probably holds true for no other illness more so than for cancer: our current understanding of this malignancy is that it is a stochastic disease, the accumulation and selection of unrepaired mutations over time, i.e., somatic mutation^14^. Generally, it is assumed that six mutations in key factors are necessary to initiate full blown cancer^15–17^, while, at least for some cancers, the need for only three driver mutations has been postulated^18^. However, cancer development is not necessarily a linear progression. Knudson, continuing from his seminal 1971 work suggests that two mutations are necessary to overcome the initial rate limiting steps^19,20^. While certain activated oncogenes can increase DNA double strand breaks and therefore the rate of mutations—the so-called mutator phenotype^21^—mathematical modelling suggests that selective pressure is sufficient to explain observed carcinogenesis^14^. Whether the increase of cancer with age (Fig. 2) can be merely reduced to cumulative acquisition of oncogenic mutations over time has been increasingly discussed and additional factors such as age-related changes of DNA methylation^22^ have also been suggested^23^. There seems little doubt that a general relationship exists between the total number of (stem) cells within a tissue, the number of cell divisions and the overall risk of developing cancer in that tissue^24,25^. Additional risk factors can significantly increase the risk of cancer by altering cell proliferation (i.e., increase the number of cell divisions), increasing the chances of mutation, or doing both, but they do not alter the underlying mechanisms at work. These risk factors can be both genetic, for example, Li-Fraumeni Syndrome, a germline mutation in the *TP53* gene, which leads to an early onset of multiple types of cancer, or environmental like cigarette smoke, which drastically increases the risk of lung cancer, still one of the most common causes of cancer-related death^26,27^.

But what does it mean when a disease closely associated with ageing occurs in children? While around 5% of all childhood cancers are due to an inherited mutation, it was long assumed that most paediatric malignancies develop due to chance^28^. With more than 1 million individual molecular lesions per cell per day^29^ and a large enough population, chance would suggest that ‘bad luck’ might lead to cancer in some younger individuals. This is supported by the fact that cancer incidences are lowest in children (Fig. 2). However, it would also mean that—ignoring cancer associated with extrinsic risk factors, e.g. lung cancer and smoking—the cancer risk for individual cancers, like ALL, should be equal in childhood and total cancer. This is not the case (Table 1). Furthermore, we know that some cancers are linked to the different stages of development: for example, early puberty (the change between a child’s and an adolescent’s body) is a known risk factor for breast cancer, while at the other end of development, aromatase blockers only work in post-menopausal breast cancer patients^30,31^. Also, looking at two consecutive age groups, children (0–14 years of age) and adolescents (15–19 years), where the additional external factors are of minimal difference, one can observe stark variations in the age-adjusted incidences of various cancers (examples are shown in Table 2). Looking at mortality, one can further observe that paediatric cancer is actually a more lethal disease (in relation to incidences) than cancer in the working-age population (Fig. 2). If this were due to children being unable to cope with doses of radio- and chemotherapy applied to adults, it would be sufficient to show that paediatric cancer and adult cancer need different treatment approaches. However, although a recent ex vivo study has suggested that paediatric organs are more primed for apoptosis than those of adults^32^, this does not reflect clinical reality. Children younger than 3–5 years of age are generally not treated with radiotherapy, but can tolerate higher doses of chemotherapy than adults. Interestingly, both treatment modalities function similarly, by the induction of apoptosis.

**Table 1:** 10 most common cancers: adults vs. children

Adults 18 + years of age (data from 2014)	Children 0–14 years of age (based on 2006–2008 average)
Breast	Leukaemia
Prostate	Brain, other CNS and intracranial tumours
Lung	Lymphomas
Bowel	Soft tissue sarcoma
melanoma skin cancer	Sympathetic nervous system tumours
Non-Hodgkin lymphoma	Renal tumours
Kidney	Bone sarcoma
Head and neck	Carcinomas and malignant melanoma
Brain, other CNS and intracranial tumours	Germ cell and gonadal tumours
Bladder	Retinoblastoma

Data source: Cancer Research UK (www.cancerresearchuk.org/health-professional/cancer-statistics), via: The data were provided by the Office for National Statistics on request, June 2016. Similar data can be found here: http://www.ons.gov.uk/peoplepopulationandcommunity/healthandsocialcare/conditionsanddiseases/bulletins/cancerregistrationstatisticsengland/previousReleases

The data were provided by ISD Scotland on request, May 2016. Similar data can be found here: http://www.isdscotland.org/Health-Topics/Cancer/Publications/

The data were provided by the Welsh Cancer Intelligence and Surveillance Unit, Health Intelligence Division, Public Health Wales on request, June 2016. Similar data can be found here: http://www.wcisu.wales.nhs.uk

The data were provided by the Northern Ireland Cancer Registry on request, May 2016. Similar data can be found here: http://www.qub.ac.uk/research-centres/nicr/

2006–2008. National Registry of Childhood Tumours/Childhood Cancer Research Group

**Table 2:** Age-adjusted cancer rates per million (for the year 2000 US standard population)

	Leukaemia	Neuroblastoma	Lymphoma	Germ cell neoplasm	Other epithelial neoplasm
0–14 years of age	49.87	10.72	16.29	5.31	6.94
15–19 years of age	31.91	0.74	50.54	28.13	49.48
X-fold difference	0.64	0.069	3.10	5.30	7.13

Data source: Siegel et al., 2014

An alternative model for oncogenesis is chromothripsis, the shattering and rearrangement of chromosomes, i.e., the replacement of the gradualism of acquiring individual mutations over time with a single catastrophic event^33^. While this hypothesis is not without controversy^34,35^, it might help to explain the development of childhood cancers. There is evidence that chromothripsis strongly contributes to Sonic-Hedgehog subtype of paediatric medulloblastoma development if a somatic *TP53* mutation is present, while evidence for chromothripsis is only detected in 13% of all medulloblastoma^36^. For neuroblastoma estimates range from 0 to 10%, and for ALL cancer initiated by chromothripsis it has been shown again to be mainly associated with a particular predisposition, in this case, the Robertsonian chromosome^37–39^. The current data therefore suggest that chromothripsis is not a mechanism that distinguishes paediatric from adult cancers. Indeed, genetic analyses show that childhood tumours exhibit very few genetic alterations compared to adult cancers^40^. While this appears initially to fit to the model of accumulating mutations over time as cause of cancer, as an explanation it is insufficient, as cell divisions do not occur uniformly over time. Indeed, most, including telomere shortening as a surrogate readout for cell division, occur mainly prior to birth^41^. Experimentally, it was shown that maximal accumulation of mutations occurs during ontogeny, the development to maturity^23^, suggesting that the few additional events, which occur later in life are needed for tipping the scale. Taken together, the data suggest that the predisposition for paediatric cancers are caused early during development, often by de novo germline mutations^42^, and that tumours arise when a combination of a few particularly aggressive mutations occurs, i.e., fewer mutations are needed than in an adult tumour, and therefore the contribution of an individual mutation must be bigger, *ceteris paribus*. This has several therapeutic implications, as follows:

(A) Children where the mutations occur early during development, either in the form of germline mutations or affecting whole tissues, are genetically distinct from children who have not developed these mutations. This might affect systemic responses to therapy.

(B) That only few mutations have occurred might also point to the possibility that paediatric tumours are genetically more stable and therefore might not as easily develop resistances to therapy as adult tumours. For some paediatric malignancies, this might contribute to the increased therapeutic successes when compared to adult tumours.

(C) The reduced number of mutations unfortunately also means there is a reduced number of tumour-specific features, which can be targeted. However, it should also be pointed out that the focus on mutations tends to ignore the fact that dysregulation, such as overexpression and deregulated activity, are important factors contributing to the oncogenic phenotype^3^. It is particularly this latter area of interest where coordinated studies are lacking.

To summarise, in paediatric oncology, both the patient, as host for the disease, and the malignancy are distinct from adults and their tumours. Therefore, any therapeutic approach trying to improve the health of childhood cancer patients must take these specificities into account. Essentially, this has already been formulated more than a century ago by another German paediatrician: “Pediatrics does not deal with miniature men and women, with reduced doses and the same class of diseases in smaller bodies, but… it has its own independent range and horizon and gives as much to general medicine as it receives from it.”^43^.

**Fig. 3:**
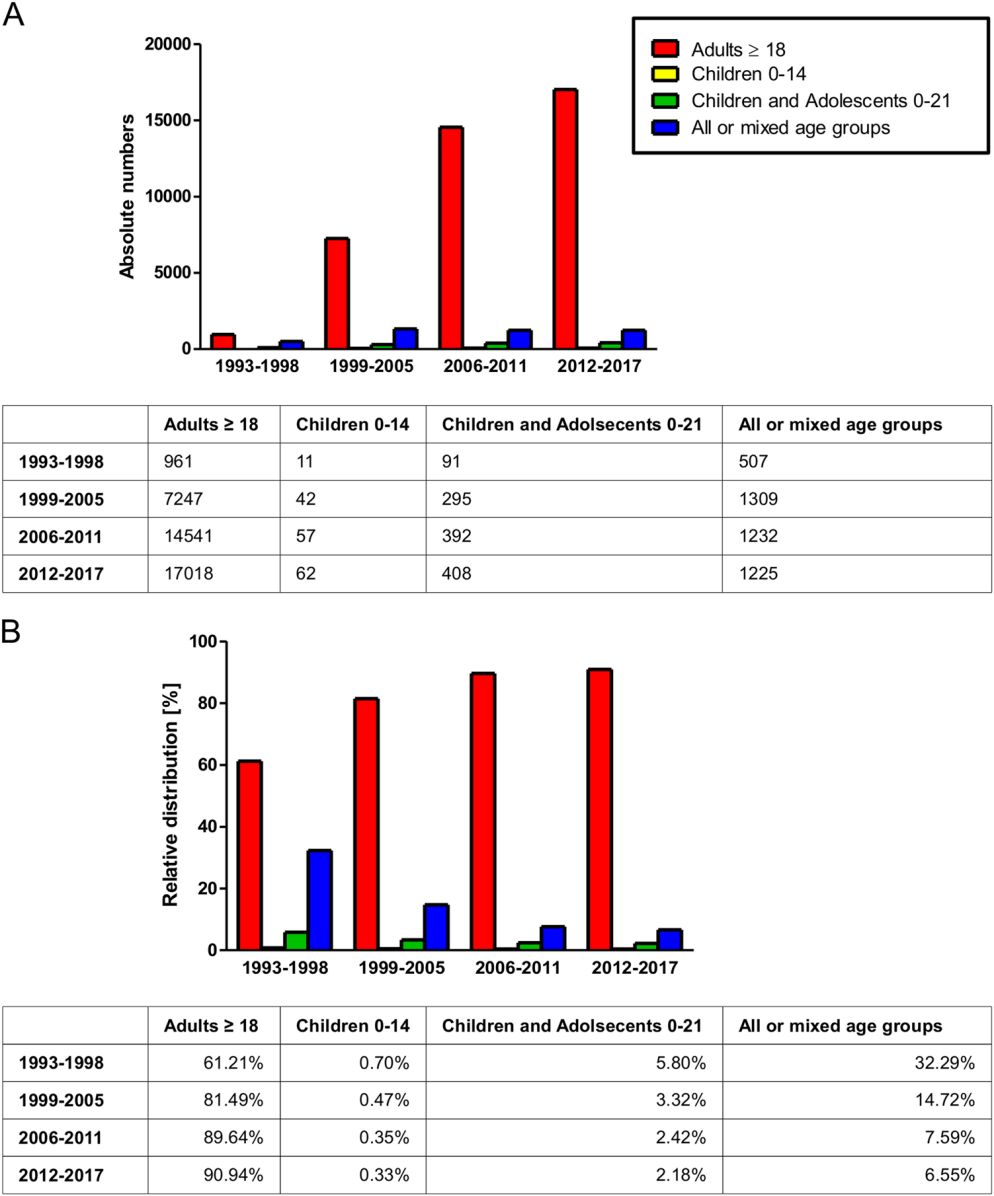
Changes over time of clinical trials grouped according to age of subjects .Interventional cancer-related clinical studies registered at ClinicalTrials.gov were categorised into four distinct groups, indicating whether the subjects were children, children and adolescents, adults or mixed (each study was allocated only one group). In **a**, absolute numbers are shown, thus indicating the development of the number of oncology studies over time, while in **b**, the distribution of the target age group of the oncology studies over time (in % of total) is depicted.Of note, the time period 1993–1998 precedes the establishment of the database and therefore only contains few appended entries, and studies without identifying starting date or with future starting date were excluded (cutoff date: 22 May 2017)

One of the key reasons why cell death-inducing treatment has been a major success story in the treatment of many childhood cancers, particularly ALL^40,44^, is that a coordinated effort was made in the 1970s by the clinics to share information and experiences, helping to standardise and further develop treatment options^3^. This success further highlights the need to treat children and childhood cancers as distinct from adults. Yet, most clinical trials are not designed specifically for children and even thosen trials that look at children do not do so exclusively, but in most cases combine them with adolescents or all age groups (Fig. 3). Partially, this is understandable, as the incidences of cancer are much higher in the adult population. However, in terms of years of life lost and common causes of death, cancer can be very well viewed as a major problem affecting children (Table 3). Currently, there seem to be two fundamentally opposite philosophical approaches that need to be resolved: *Protecting children from clinical trials* (by extrapolating data from adult trials) vs. *Protecting children with clinical trials* (by finding the optimal therapeutic approach). The authors, both clinicians/researchers and parents/grandparents, are well aware that resolving this conflict is neither easy nor can it ever be fully satisfying or without considerable emotional burden.

**Table 3:** Cancer ranked as leading cause of death, the absolute number of deaths and the average years of life lost for the ten age groups of the US American population 2006

**Age group:**	<1	1–4	5–9	10–14	15–24	25–34	35–44	45–54	55–64	65+
Rank as cause of death	>10	3	2	2	4	4	2	1	1	2
Absolute numbers	n/a	317	459	448	1664	3656	13917	50334	101454	387515
Year of life lost	n/a	23775	31946.4	28492.8	92019.2	160132.8	432818.7	1016746.8	882649.8	n/a

Total number of incidences:		Total years of life lost:	
Childhood cancers	1224	Childhood cancers	84214.2
Non-childhood cancers	558540	Non-childhood cancers	2584367.3
Total	559764	Total	2668581.5
Relative contribution of childhood cancers	0.22%	Relative contribution of childhood cancers	3.16%

Data source: National Vital Statistics System, National Center for Health Statistics, CDC and the World Bank

## Long-term considerations

*Caedite eos! Novit enim Dominus qui sunt eius* already indicates that destroying a population is not possible without collateral damage. In adult cancer, we see this predominantly in the short-term complications of treatment, such as fatigue, diarrhoea, nausea, vomiting and hair loss, as well as neuropathy, anaemia and thrombocytopenia. In paediatric oncology, one also has to consider the long-term implications, as successful treatment hopefully means the patient has another three score and 10 years to live. In a recent review discussing radiation and brain tumours we identify the following complications, which might arise from using cell death-inducing treatment in children: A link has been postulated between the exposure of children to radiotherapy for leukaemia and the overall risk of developing glioblastoma^45^. Even computed tomography (CT) scans, needed to optimise targeting and monitoring therapy success, are associated with increased risk of brain cancer^46^. Childhood cancer survivors have increased risks for several malignancies later in life such as Acute Myeloid Leukaemia (AML), Non-Hodgkin lymphoma and colorectal cancer^47,48^. Overall, the risk of developing a second cancer is 3- to 6-fold higher in survivors of childhood cancer than in the general population^49^. Importantly, not only radiation, but also various forms of chemotherapy are known risk factors^48,49^.

Already in 1969, Bloom and co-workers demonstrated cognitive dementia among survivors of childhood medulloblastoma^50^, and while some earlier studies suggested that the tumour might be the causative agent for reduction in IQ, most large-scale studies agree that radiotherapy is an independent risk factor. Importantly, younger age of patient at treatment, radiation dose and time since treatment enhance the neurological deficits^51^. This might suggest that radiotherapy damages the pool of neurological stem cells, replenishment becoming more and more inefficient with increasing age, and thus neurological deficits increasing. This hypothesis is supported by the observation that conformal avoidance of the hippocampal neural stem cell compartment reduces neurological side effects^52^, and hyperfractionated radiotherapy, at doses of 1 Gy, can also prevent neurological decline, without the loss of therapeutic efficacy^53^. However, radiotherapy is also associated with additional side effects in childhood brain cancer survivors, such as hypothalamic-pituitory dysfunction and hearing loss^54,55^.

While radiation, by the nature of localised application, has rather specific side effects, often relating to the tumour-harbouring organs, chemotherapy is given systemically and therefore a wide range of side effects is to be expected. Indeed, here we find growth, intellectual development and entry into puberty being delayed, as well as reduced fertility and organ-specific damage, mainly to heart and lungs^56^. Importantly, the original damage caused by toxicity is often unrecognised or subclinical at the time and only during development, when the tissue is exposed to further stress, do the long-term side effects become apparent^56^.

In the recent decade, the large data sets on long-term survivors of several paediatric cancers became available, as a direct result of the successes in the 1970s. Previous data were mainly collected from predominantly benign tumours, like medulloblastoma, but now children also survive more aggressive tumours, partially due to more aggressive treatment. It is in the very nature of long-term side effects that our knowledge will always be lagging behind by a few decades and as long as we have to consider undetectable organ damage, we need to remain vigilant.

The aim of cell death-inducing therapies in paediatric oncology must be two-fold: In the large group of cancers, which we can successfully treat, we must minimise the severe, long-term side effects that occur, while for those tumour entities that remain therapy-resistant, we must improve our therapeutic choices. Precision medicine potentially addresses both those aims.

## Precision medicine: chances and perspectives

Personalised medicine, i.e., the tailoring of the therapeutic intervention towards the individual cancer (and not actually the person), is—as such—not a new approach in paediatric oncology: already the early protocols were stratified according to risk groups, originally according to patient’s age and gender, but also cells of origin: for example, it has long been known that a T-cell ALL has a worse prognosis than a pre-B-cell ALL^3^. This was later augmented by cytogenetic analyses, looking at different chromosome content and composition; in ALL the presence of the so-called Philadelphia Chromosome (resulting from a t(9;22) translocation) calls for a particularly aggressive treatment. With the advent of high throughput and deep sequencing, we now have increasing data on the molecular composition of individual cell populations in the malignancies and the challenge has shifted to extracting therapeutically relevant information. This will mean both effective data mining techniques and the mapping of in silico information onto cellular processes. The conflict of how best to achieve this is reflected in the famous debate between Weinberg and Golub^57,58^. The INFORM study and the NCI-MATCH trial only identified targetable genetic abnormalities in around 10% of paediatric patients^59,60^. The equivalent studies looking at adult cancer have further shown that personalised medicine leads to no improvement over “doctor’s choice”^61,62^.

**Fig. 4:**
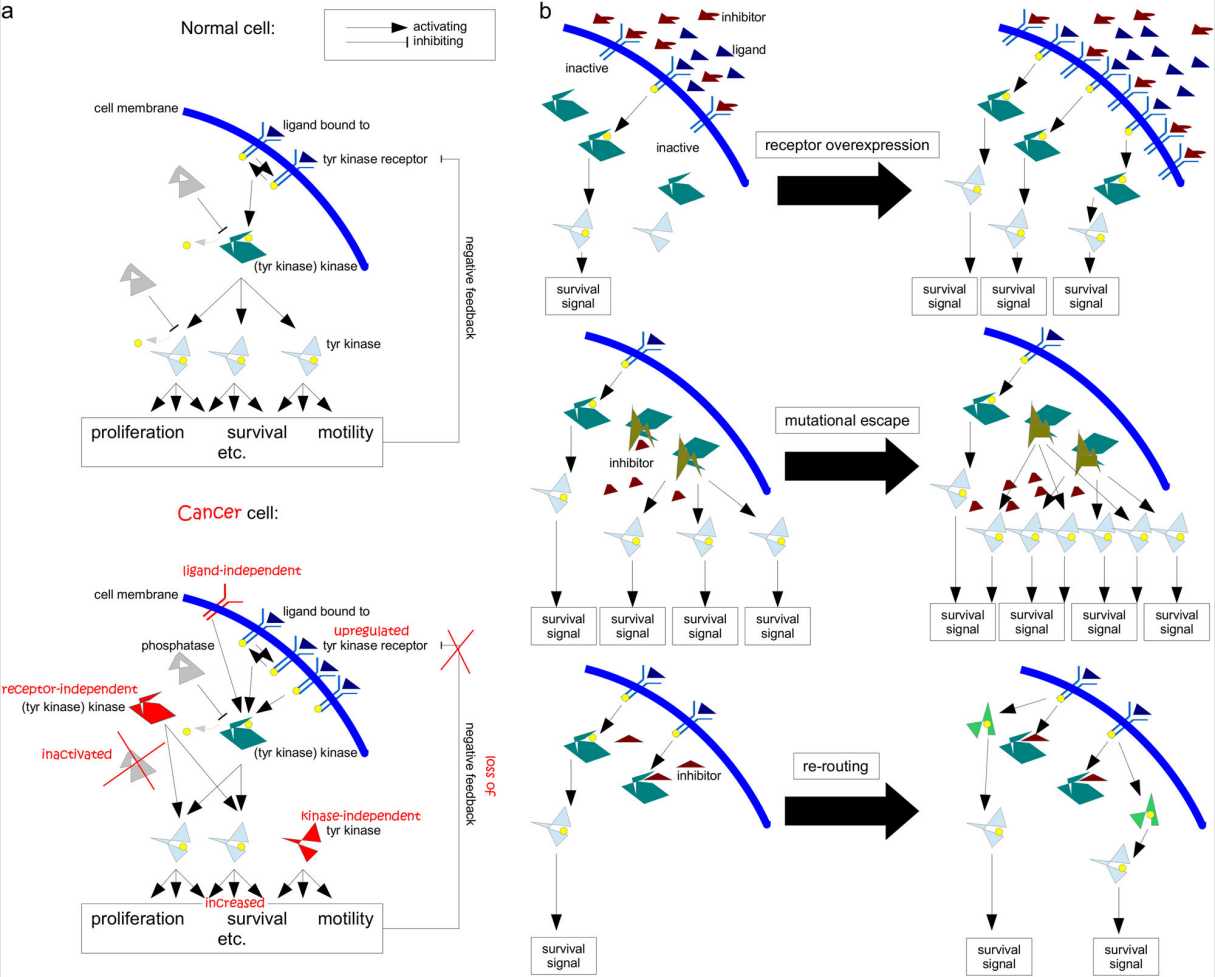
Target structures for pharmacological inhibitors and common escape mechanisms **a** Aberrant activation of tyrosine kinases as a mechanism for malignant transformation. Cancer cells are defined by overactive signalling cascades, often mediated by tyrosine (tyr) kinases. Common therapeutic strategies are either blocking of the tyr kinase receptor by inhibiting antibody/pharmacological inhibitor (which does not work for ligand-independent signals and has reduced potency if the target is overexpressed), or utilising pharmacological inhibitors that block kinase activity (dependent/independent of mutational status). Modified from a previous version^3^. **b** Three common escape mechanisms from kinase inhibition. The three common mechanism by which tumour cells escape the blocking effect of small molecule inhibitors are (from left to right): (I) Upregulation of the target structure, for example the tyr kinase receptor. This can occur independently of any genetic alterations and is potentially reversible, i.e., after discontinuation of treatment the tumour can become re-sensitised (as seen, for example, in AML^89^). (II) Mutation of the target structure, so that the inhibitor can no longer bind. This is frequently observed during monotherapy, where only one new selective pressure is applied. For example, initial reports on the novel tyrosine kinase inhibitor imatinib indicated that treatment restored the life expectancy of chronic myelogenous leukaemia patients to that of the general population^90^. While imatinib is a potent drug that greatly improves patients’ quantity and quality of life, treatment-resistant cancer cells frequently emerged and prevented a complete cure^91^. (III) Due to the interconnectedness of signalling cascades, inhibition of specific survival molecules can lead to a re-routing or re-wiring of the signals via alternative intermediates, which can result in the formulation of the Nile Distributary hypothesis, that postulates that some cancers are particularly aggressive as they use “multiple cross-covering growth enhancing pathways to grow and avoid cytotoxic interventions”^92^

The current approach is implicitly linked to our understanding of how tumours arise, accumulating gain-of-function mutations (proto-oncogene to oncogene) and detrimental mutations (tumour suppressor inactivation) to acquire the hallmarks of cancer, which allow the tumours to grow independently of external control^63,64^. This is mainly mediated by kinases, predominantly tyrosine kinases (Fig. 4a). Identifying the mutations in the individual tumours indicates which pathways are abnormally activated and targeting these with pharmacological inhibitors should to a certain extent restore ‘normality’, e.g., reduce apoptosis resistance and make the tumour more amenable to cell death-inducing therapies. While the logic of this strategy is sound, there are several key factors, which might explain why the clinical application of this strategy has so far not been overly successful:

1. The complexity of the signalling networks is far greater than usually admitted. While signalling pathways are generally described in linear terms with defined functions, in reality they are more akin to a circuit board with almost infinite intricacy^64^. The PI3K/Akt/mTOR cascade, for example, is highly interwoven with the MEK/Erk signalling pathway, interacting, both in an inhibitory, as well as an activating function, at multiple points and influencing cellular behaviour with respect to survival, cell cycle progression, metabolism, motility and DNA repair^10,65^. Our own research has highlighted the complexity of finding the optimal combination of inhibitor and cell death-inducing agent. Combing a dual inhibitor of PI3K and mTOR with the chemotherapeutic agent doxorubicin can have very distinct effects on neuroblastoma cells depending on the sequence of application, where inhibiting PI3K signalling after inducing apoptosis leads to a higher cell death rate than blocking it prior to the addition of doxorubicin, i.e., maximal inhibition of a survival cascade does not necessarily lead to maximal therapeutic benefits^66^. This was also confirmed by looking at the RIST therapy, a complex combination therapy that combines two chemotherapeutic agents with two pharmacological inhibitors in a compassionate use setting^67^. In an attempt to improve efficacy, When replacing rapamycin, an mTOR inhibitor, with GDC-0941, which blocks PI3K, i.e. acts more upstream in a glioblastoma cell culture system, this alternative RIST approach showed higher potency, but upon transferring it to a murine system all potency was lost. It appears that this was due to GDC-0941 reducing tumour vascularisation and, thus, minimising repeated delivery of the chemotherapeutic agents^67^. In essence, the inhibitor worked less well in combination because it worked better as a single agent. Translating these emerging pharmacokinetic and -dynamic interactions into the human system is going to be one of the major challenges of modern combination therapy.
2. In personalised medicine the target is always selected by frequency, i.e. mutations found in the most common populations of cancer cells are detected and selected as targets. However, at initiation of treatment the tumour might contain 200,000 to 2,000,000 undetected cells that are distinct from the dominant subclone^1^. The most common population within a cancer is obviously the best adapted one (to the current microenvironment), but, of course, by initiating treatment we change the environment and create new selective forces. We have discussed the complex Darwinian mechanisms occurring in a tumour elsewhere^1,10,65,68^, but if one transfers Luria and Dellbrück’s seminal work from 1943 to the evolution of cancer, it would suggest that any precision therapy will lead to the emergence of a treatment-resistant disease^69^. There are computer models that devise a treatment strategy for bacteria so as to avoid antibiotic resistance^70^. On the basis of a similar sequential approach, we showed enhanced treatment efficacy in neuroblastoma^66^. We need to understand which tumour cells exist that might already be resistant to the treatment and which preferred routes of acquired resistance might be taken. This is also important as targeting a tumour-specific aberration can lead to emergence of rapid resistance via several possible routes (Fig. 4b).
  In essence, a precision + approach is needed (Fig. 5), specific enough to minimise damage to the host, and broad enough also to target both minor and likely future cell populations. To quote Emerson again, “[w]hen you strike at a king, you must kill him”. This certainly holds true for the emperor of all maladies - once the decision is made to cure the cancer and not chronify it preventing the emergence of a recessive disease should be our primary aim^1,10,65^.

**Fig. 5:**
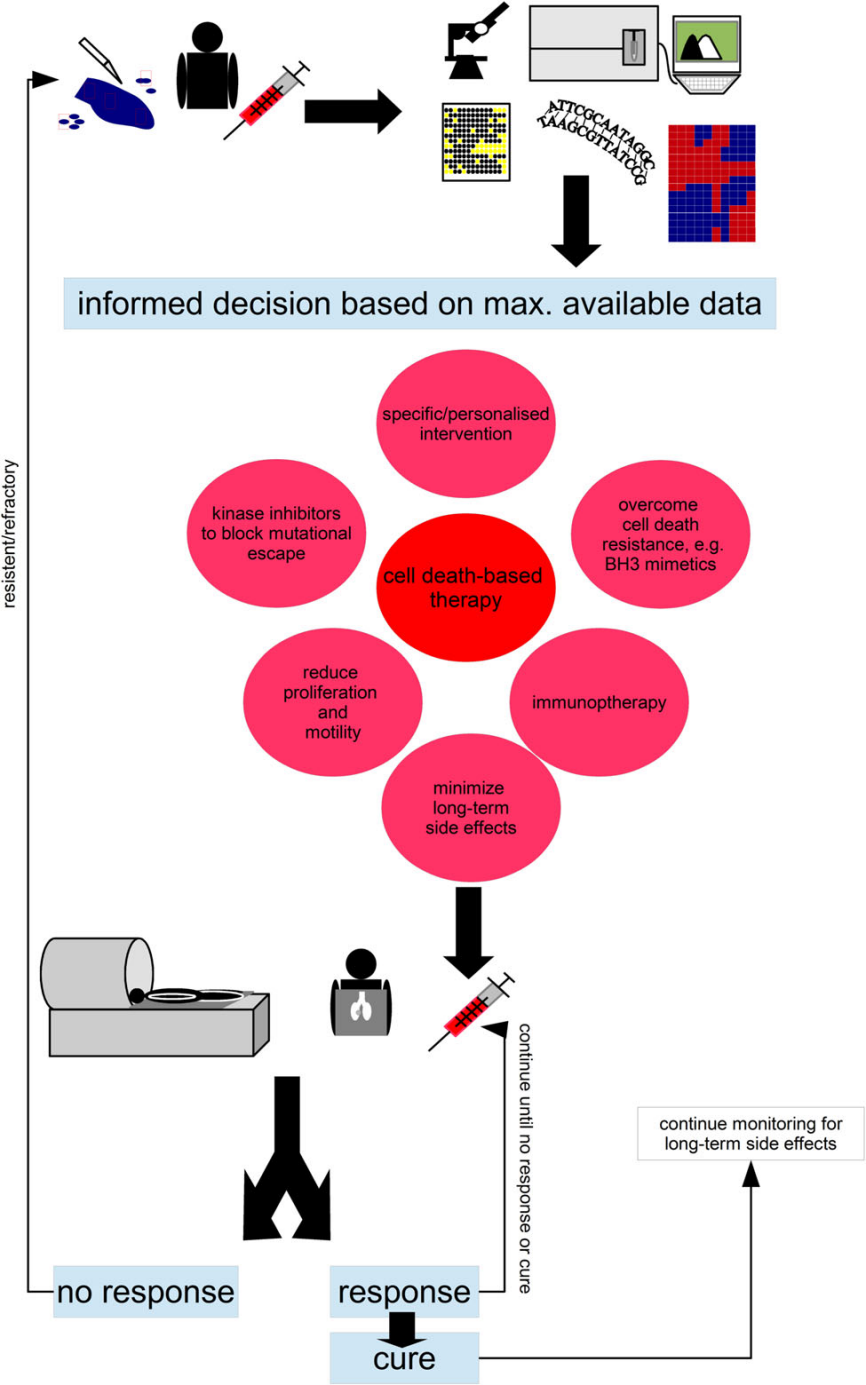
Precision+Therapy Collect as much data on the (epi)genetic make-up of the malignancy, its potential satellites and the patent. On the basis of somatic mutations, expression patterns, phosphorylation and levels of proteins as well as clinical knowledge devise a therapeutic intervention.Important questions that need answering: How much sampling is needed to get a comprehensive picture of the ecological niche and the various subclonal populations that make up the cancer? How best to identify the data that is of therapeutic relevance, tumours in adults can present >10,000 genetic alterations?^93^ The centre piece of any precision therapy will be cell death-based treatment, to reduce tumour burden, combine this pharmacological molecules that target tumour-specific alterations, drugs that overcome apoptosis resistance such as BH3 mimetics (as discussed in Fig. 6) and substances that minimise cell proliferation (no full blockage as many therapeutic interventions are dependent on proliferation) and block invasion, as to avoid ‘the cost of migration’ or ‘go or grow’: it has been shown that the lack of oxygen can induce genes that are responsible for motility, which, in turn, reduces proliferation^73,74^. Similarly, epithelial–to–mesenchymal transition which is most linked to invasion and several surface molecules, which also mediate adhesion should be prevented^10,94^. In addition, add additional inhibitors to prevent the emergence of resistance mechanisms as discussed in Fig. 4b. The patient’s immune system should also be trained to further sponge up cells which have escaped the initial therapeutic intervention, here modern immunotherapy holds several promises^3^. Finally, known side effects can also be countered at this stage already. For example, neurocognitive impairment, i.e., cognitive dementia, associated with radiotherapy can be reduced by neurological and pharmacological interventions^95–97^. Important questions that need answering: How predictable are the emerging resistance patterns, will blocking certain routes just provoke evolutionary escape via other mechanisms without greatly improving therapeutic outcome? Which sequence of administration should be chosen and how to further optimise this? The patients need to be closely monitored and any changes that suggest mutational escape of the tumour will need to fully restart the precision + therapeutic protocol. Even upon cure monitoring needs to be continued to identify long-term side effects, such as the emergence of secondary cancers, as early as possible. Important questions to ask: How to best monitor patients, as many radiation-based screening methods are themselves associated with increased cancer risks?^46^ Which screening methods should be employed for cured patients, as the long-term side effects have proven to be hard to detect and will be extremely varied for a multi-modular therapy. Importantly, the free and frank exchange between the paediatric oncology community which was the basis for the successes of the 1970s must be sustained throughout every step
3. The choice of target also requires some consideration. Obviously, an early initiating mutation will be present in most, if not all, cancer cells, but there is no reason to assume that a genetic alteration necessary for oncogenesis is a good target to sensitise cells for treatment. Indeed, several tumour-specific alterations seem to have multiple contradictory functions in a tumour, for example in Ewing Sarcoma the fusion protein EWS/ETS and other proteins overexpressed in this tumour, such as DKK2, contribute negatively to proliferation, but enhance metastasis^71,72^. A similar relationship has also been observed in glioblastoma, where it is called ‘go or growth’ or ‘the cost of migration’: increased tumour invasion is linked to reduced proliferation^73,74^. In glioblastoma, we find a high incidence of MGMT promoter methylation, which is assumed to allow for higher tumour flexibility by increasing the rate of (unrepaired) mutations. However, while it also sensitises the tumour to treatment with temozolomide (a radical change in environment), promoter methylation strongly correlates with overall survival (independent of treatment) in paediatric glioblastoma^75^. Therefore, treating glioblastoma with temozolomide, while improving survival rates by a few months^76^, also selects for tumour cells with worse prognosis. Therefore, while mutated gene products or fusion proteins are an obvious and highly specific target, they could have several, competing functions that do not make them ideal targets. Using components within combination therapy that are closely and (as far as we know) fairly specifically associated with apoptosis resistance might be a promising alternative such as targeting the Bcl-2 family or IAPs (Fig. 6).

**Fig. 6:**
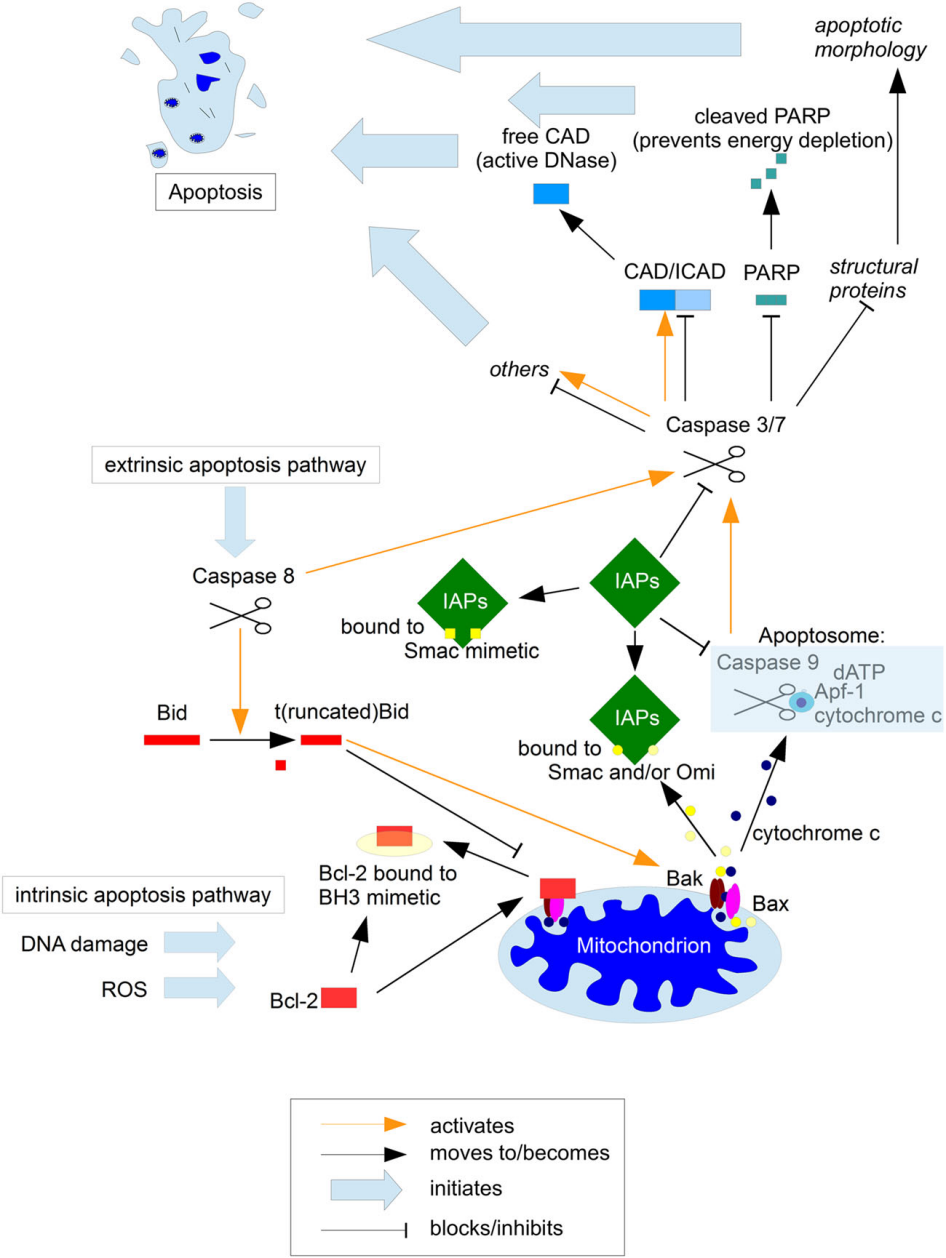
The roles of the Bcl-2 family and IAPs in apoptosis regulation Apoptosis is a highly complex and tightly regulated cell death program^10^. In recent years two classes of proteins have come increasingly into the focus of potential therapeutic interventions, the Bcl-2 family and IAPs. The Bcl-2 family (red) comprises proteins that contain at least one Bcl-2 homology (BH) domain and fall into three subgroups: the BH3-only, the pro-survival and the proapoptotic proteins. Cellular stress stimulates BH3-only proteins which then alter the balance between pro- and anti-apoptotic Bcl-2 family proteins leading to BAX and BAK-forming pores and thus initiating mitochondrial apoptosis^65^. The most promising approach with regards to this protein family are BH3 mimetics that have a function similar to that of BH3-only proteins. While BH3 mimetics on their own can be sufficient to induce apoptosis, generally some form of additional stress is needed for optimal efficacy^65^. Interestingly, in a preclinical setting, successes with BH3 mimetics could be achieved particularly in the high-risk groups of ALL and neuroblastoma^3^. It is tempting to speculate that within particularly aggressive tumours, be it due to the high proliferation rate or the fact that invasive cells more frequently encounter a hostile environment, the intrinsic cellular stress is already at a higher level, so that less extrinsic force is needs to be combined with the mimetics. While the Bcl-2 family contribute to apoptosis resistance directly prior to/at the mitochondria, there is a second class of proteins (green) that mediate cell death resistance at the stages following mitochondrial involvement. Of the eight mammalian Inhibitor of Apoptosis Proteins (IAPs), three are ubiquitously expressed, XIAP, c-IAP 1 and 2^65^. XIAP binds and thereby inhibits Caspases-3, -7 and -9, while the role of the c-IAPs seems slightly more complex; while there is considerable functional overlap with XIAP, they also have a crucial role in RIP1-dependent necrosis or necroptosis^98,99^. Therapeutically, mimetics of the naturally occurring IAP inhibitor, Smac/DIABOLO have shown great promise. Unlike BH3 mimetics which can either sensitise for, or induce cell death in a tumour-dependent manner^65^, Smac mimetics are generally considered as sensitisers and not inducers of apoptosis^99^. Their potency was first demonstrated using an orthotopic human glioblastoma model, where the combination of Smac mimetic and death ligand Apo2-L/TRAIL *de facto* cured the mice, while the individual substances had no or little effect on overall survival^100^

An enhanced focus on these two groups might also reduce a risk associated with the use of kinase inhibitors. Many of these kinases associated with cancer also play a critical role in development and therefore will cause a distinct set of side effects in children compared to adults^77^. Currently, only a very limited number of targeted drugs are approved for the treatment. As this will have to change in future more work is needed to understand and counter these potential side effects.

It had long been hoped that in addition to the common cell death-inducing therapies, the induction of apoptosis via triggering death receptors, such as Apo1/CD95 and Apo2/TRAIL-R, might prove a feasible approach with less severe side effects^78^. However, two findings have rather impeded the development of death rector triggering as treatment: (1) the discovery of pro-tumourigenic properties of these receptors and their ligands^79–81^ and (2) the fact that paediatric cancers, such as neuroblastoma and primitive neuroectodermal brain tumours, often exhibit resistance to death receptor-triggered apoptosis via downregulation of Caspase-8^82,83^. Importantly, the clinical evaluation of Apo2-L/TRAIL has also been disappointing, while for CD95 an antagonist, APG101, has been shown to be clinically beneficial for glioblastoma patients^84,85^. Death receptors remain an interesting potential target for cancer therapy; however, there is still considerable work needed to successfully integrate their triggering into a complex therapeutic regiment^84^.

## Conclusions

If we want further to improve the cure rate of patients with paediatric malignancies several factors have to be considered. Neither host/patient nor disease are identical to post-pubescent individuals and their disease. While adult oncology will still be needed to inform and initiate novel therapeutic approaches, we will need to further our understanding of the nature of paediatric cancers and how they react to treatment.

Essentially, any therapeutic approach that aims to cure the patient will need to be built around cell death induction. However, in paediatrics, we have to be much more aware of the long-term consequences, be they developmental or the risk of treatment induced secondary malignancies. The introduction of metronomic chemotherapy protocols a decade ago, implementing the continuous or more frequent administration of lower therapeutic doses, has already lowered the risks of acute side effects. Moving away from monotherapies to increasingly complex combination therapies, an approach that was also pioneered in paediatric oncology before it was taken up for the treatment of adults, will further prevent mutational escape. We already have a considerable arsenal of kinase inhibitors for use in paediatric oncology (Table 4), and combining several of these into a cocktail with cell death inducing therapy and BH3 mimetics will open new avenues of intervention. Here, the challenge lies in understanding the complex interactions of signalling networks in malignant cells to allow the use of the optimal sequence of the optimal combination, personalised for both patient and disease.

**Table 4:** Kinases and their inhibitors in select paediatric tumours

Genomic alteration	Target structure	Medication	Example pediatric tumour
Loss of PTEN PIK3CA mutations	PI3K/mTOR	Everolimus Temsirolimus Rapamycin	Sarcoma
N/KRAS mutation PTPN11 mutation	MEK	Trametinib Selumetinib	Melanoma glioblastoma juvenile myelomonocytic leukaemia
BRAF mutations/ fusions	BRAF	Vemurafenib Dabrafenib	Melanoma Langerhans-cell histiocytosis glioma
ALK mutation/fusion	ALK	Crizotinib	Neuroblastoma embryonal tumours
PTCH1 mutation	SMO	Vismodegib	Medullablastoma
BRCA1/2 mutations EWSR1-FLI fusion ATM mutation	PARP1	Olaparib Rucaparib	Osteosarcoma Ewing sarcoma
Biomarker MYCN amplification	AURKA	Alisertib	Neuroblastoma
FGFR1/2/3 fusion, amplification, mutation	FGFR	Ponatinib Dovitinib	Rhabdomyosarcoma Ewing sarcoma
FLT3 mutation or internal tandem duplication	Target multikinase inhibitor	Sorafenib	Acute myeloid leukaemia
VEGF Receptor expression of cKit and PDGF Receptor	Multikinase inhibitor	Pazopanib	Sarcoma

Data source ref.^40^

Another treatment approach which should be combined with this complex combination therapy is immunotherapy, using both the patient’s innate immune system and adaptive immunity to kill cancer cells. Here several forms of medication are also already available (Table 4).

It has been estimated that in 1980 0.1% of all 20-year-old US Americans was a childhood cancer survivor^86^, yet paediatric malignancies are still a leading cause of death among children. We, therefore, need a coordinated effort both to understand long-term consequences of our treatments and to improve therapeutic efficacy. This information will have to come from a frank and free exchange of clinical information, i.e., the continuation of a successful approach initiated in the 1970s, and clinical trials. Here, allowing for the lower overall number of cases, we must rethink our trial designs and incorporate rolling six designs and continuous recruitment^87,88^.
